# Common Pregnancy Complaints Can Lead to Motor Vehicle Collisions or Near-Miss Incidents

**DOI:** 10.3390/healthcare10020279

**Published:** 2022-01-31

**Authors:** Sachi Tsuchikawa, Kyoko Hanahara, Yumiko Tateoka, Masahito Hitosugi

**Affiliations:** 1 Department of Nursing, Shiga University of Medical Science, Otsu 520-2192, Japan; pom1121@belle.shiga-med.ac.jp (S.T.); ytateoka@belle.shiga-med.ac.jp (Y.T.); 2 Faculty of Nursing, Seisen University, Hikone 521-1123, Japan; hanaha-k@seisen.ac.jp; 3 Department of Legal Medicine, Shiga University of Medical Science, Otsu 520-2192, Japan

**Keywords:** driving, pregnant woman, common complaints in pregnancy, motor vehicle collision, near-miss incident, safety, health guidance

## Abstract

Pregnant women commonly report various health complaints during pregnancy, the occurrence of which is believed to cause human error. However, no study has examined the relationship between the occurrence of pregnancy complaints and the risk of motor vehicle collisions (MVCs). This study aimed to clarify the relationship between the frequency and severity of common pregnancy complaints and the occurrence of MVCs or near-miss incidents. We conducted a multicenter cross-sectional survey of 1000 pregnant women in Shiga Prefecture, Japan. The event group experiencing MVCs or near-miss incidents during pregnancy comprised 10.8% of respondents. The frequency of compression of the stomach or abdomen, tension and cramps in the lower abdomen, pelvic pain, irritability, depressed mood, distractedness, and hot flashes was significantly higher in the event group. The results of our multivariate logistic regression analysis revealed that tension and cramps in the lower abdomen, distractedness, and irritability were independent contributory factors to such events, with an odds ratio of 2.414, 1.849, and 1.746, respectively. Educating pregnant women to avoid driving when experiencing these symptoms would improve maternal and fetal safety.

## 1. Introduction

One of the 2030 Agenda development goals adopted by the United Nations Member States in 2015 is a healthy life for all and the promotion of well-being for all, at all ages. More specific goals include reducing the global maternal mortality rate, reducing mortality in newborns, and reducing mortality in children under the age of 5 years [1]. Recently, low birth rates have become a huge problem in developed countries. In Japan, the number of children aged 15 years or under has decreased over the past 31 years owing to a continuously low birth rate. Japan had a total fertility rate of 1.42 in 2018, lower than those of France (1.88), the United States (1.73), and England (1.68) [2]. Therefore, protecting the life of all children, including unborn fetuses, is of the highest priority, especially in Japan.

A population-based, self-matched, longitudinal cohort analysis of the risk of motor vehicle collisions (MVCs) involving pregnant women drivers in Ontario suggested that the risk of a serious MVC significantly increased during the early second trimester of pregnancy [3]. Although the authors did not identify the reason for this increase, psychological and physiological changes during pregnancy were considered contributory factors. A survey of pregnant women suggested that more than half of passengers who did not always use a seatbelt complained of discomfort [4]. According to a survey of pregnant women drivers, 43.3% suffered from compression of the abdomen while driving, and 83.9% preferred to sit on a soft seat to reduce the compression [5]. This indicates that pregnant women drivers became more sensitive when sitting in the vehicle during pregnancy than they had been beforehand.

Pregnant women often suffer from nausea, vomiting, severe drowsiness, and back pain due to the secretion of human chorionic gonadotropin (hCG) and the enlargement of the uterus. These symptoms are considered common complaints of pregnancy, and their appearance is attributed to the occurrence of human error [3]. However, although the most common causes of MVCs involve driver human error, no studies have examined the relationship between common complaints in pregnancy and the risk of MVCs.

This study aimed to address this research gap by clarifying the relationship between the frequency and severity of common complaints in pregnancy and the occurrence of MVCs or near-miss incidents, and to propose effective measures for preventing MVCs during pregnancy.

## 2. Materials and Methods

### 2.1. Study Design

This study adopted a multicenter, cross-sectional cohort design.

### 2.2. Study Participants 

**Figure 1:**
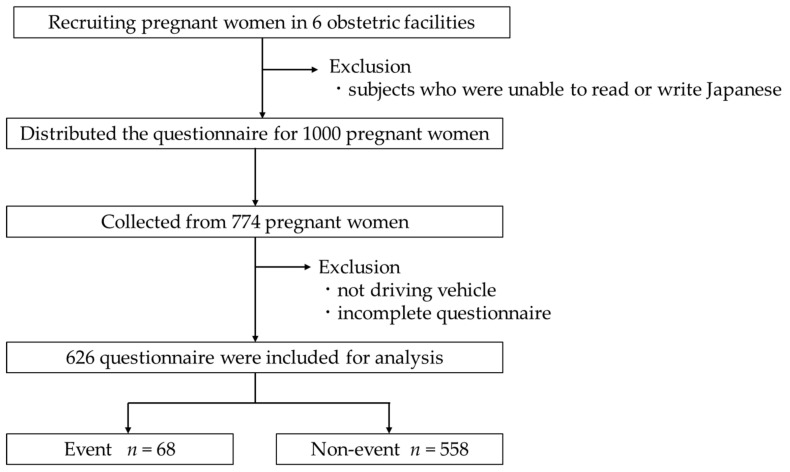
Overview of study subjects.

The study participants comprised 1000 pregnant women who visited six obstetric facilities in Shiga Prefecture from August to December 2018. This study targeted pregnant women outpatients. Exclusion criteria were the inability to read and write in Japanese (Figure 1).

Questionnaires were collected from 774 pregnant women (77.4% of the subjects). However, 148 questionnaires were excluded because they were incomplete and because the respondents did not drive a car on a daily basis. Finally, data from 626 pregnant women were included in the analysis. The women who had experienced MVCs or near-miss incidents were classified as the event group and those who had not were classified as the non-event group. Each variable was compared across the two groups.

### 2.3. Survey

After requesting and obtaining permission from each obstetrics clinic and the hospital staff, a self-administered questionnaire was distributed to the pregnant women during maternal health check-ups at each facility. Pregnant women were asked to participate in this research after reading the information sheet, which explained the purpose and duration of the study and guaranteed participants’ anonymity. The sheet explained that the submission of the completed questionnaire constituted the provision of consent. The pregnant women who consented to participate in this study were asked to complete the questionnaire and drop it in a box in the reception area of each facility. Therefore, consent was considered obtained when participants’ completed and submitted the survey. The Research Ethics Committee of the Shiga University of Medical Science approved this study (No. 29-245).

### 2.4. Questionnaire Items

The following items were included in the questionnaire.

(1) Participant characteristics including age, height, weight, and body mass index (BMI).
(2) Information about pregnancy: primipara or multipara, and gestational age.
(3) Driving history.
(4) Experience of an MVC or near-miss incident during pregnancy.
(5) Frequency of common complaints in pregnancy. We asked participants to report their awareness of the following 18 items using a four-point scale (nothing: 1, almost none: 2, occasionally: 3, always: 4): compression of the stomach or abdomen; nausea; frequent urination; tension and cramps in the lower abdomen; taut or tingling breasts; stiff shoulder; pelvic pain; back or lower back pain; fatigue and cramp in the lower limbs; whole body malaise; strong drowsiness; irritability; depressed mood; distractedness; feeling of weakness; palpitations; hot flashes; itching.

### 2.5. Statistical Analysis

Data were summarized in the form of values with proportions or frequencies for categorical variables. After confirming normality, we used the median to summarize continuous variables and the interquartile range (IQR) for values that did not follow a normal distribution. Chi-square tests were used to compare prevalence between the two groups. A Mann–Whitney test was conducted for values without a normal distribution to identify the differences in values between the two groups.

A multivariate logistic regression analysis was performed to identify the independent variables that influenced each event. The stepwise method was applied to adjust for potential confounding factors. The analysis was performed using SPSS version 25 for Windows. A *p* value of less than 0.05 was considered statistically significant.

## 3. Results

### 3.1. Basic Subject Details

The median age with IQR of all subjects was 31.0 (28.0–35.0) years, their median height was 159.0 (155.0–162.0) cm, their median pre-pregnancy weight was 51.0 (48.0–56.0) kg, and their median gestational age in weeks was 27.0 (20.0–33.0). In total, 19 pregnant women (3.0%) had experienced MVCs and 49 (7.8%) had experienced near-miss incidents. Therefore, 68 pregnant women (10.8%) were included in the event group and 558 (89.0%) were assigned to the non-event group. First, participants’ backgrounds were compared across groups. No significant differences were found between the two groups across all items (Table 1).

### 3.2. Comparison of the Frequency of Each Common Complaint in Pregnancy between the Two Groups

Participants were asked to report the frequency of awareness of common complaints in pregnancy. The answers “none” or “almost none” were classified as “low frequency”, while “occasional” or “always present” were classified as “high frequency”. In the event group, the frequency was significantly higher for six of the 18 items: compression of the stomach or abdomen, tension and cramps in the lower abdomen, pelvic pain, irritability, depressed mood, and distractedness (Table 2).

### 3.3. Relationship between Event Experiences and Common Complaints in Pregnancy

Multivariate logistic regression analysis was conducted using the items that differed significantly following the comparison between the two groups. We believed that these six symptoms influenced pregnant women’s driving ability. Therefore, we selected six items as independent variables: “compression of the stomach or abdomen”, “tension or pain in the lower abdomen”, “pelvic pain”, “irritability”, “depressed mood”, and “distraction”. As a result, “tension and cramps in the lower abdominal”, “distractedness”, and “irritability” were selected as independent factors for the event, with odds ratios of 2.414, 1.849, and 1.746, respectively (Table 3).

## 4. Discussion

In Japan, no comprehensive statistics on MVCs involving pregnant women have been collected. In one survey in Japan, 2.9% of pregnant women reported having suffered an MVC during their current pregnancy [6]. However, the prevalence of near-miss incidents had not been previously reported. The present study confirmed that the prevalence of MVCs and near-misses in the study sample was 3.0% and 7.8%, respectively, which together accounted for 10.8% of the respondents. This result was consistent with previous data collected from professional drivers, showing that 10.1% of drivers suffered from MVCs or near-miss incidents due to health problems [7]. Therefore, the effects of pregnancy-related health conditions are similar to acute health changes experienced by non-pregnant drivers. According to a previous study, around half of the pregnant women involved in MVCs experienced premature placental abruption, imminent preterm delivery, or fatalities [8]. Therefore, complications related to MVCs frequently require urgent obstetrical intervention. Furthermore, the results of the current study indicated that considerable numbers of pregnant women experienced near-miss incidents. These women also suffered from acute psychological changes, such as fear, anxiety, and sometimes surge activation of the sympathetic nervous system. Therefore, psychological care or counseling is desirable for pregnant women drivers who have experienced near-miss incidents. The results of the current study indicate that interventions by healthcare professionals to alleviate common complaints in pregnancy are urgently required.

To date, although drivers’ health changes have been considered an influence on driving behavior, there have been no reports on the common complaints of pregnant women and their influence on driving behavior. Therefore, we first confirmed that common complaints in pregnancy occurred more frequently in the event group. The frequency of nausea, tension, and cramps in the lower abdomen, pelvic pain, severe drowsiness, irritability, depressed mood, distractedness, and hot flashes was significantly higher in the event group. These findings were probably linked to the following factors. The secretion of hCG during early pregnancy, as well as mechanical compression caused by enlargement of the uterus during late pregnancy, can cause nausea and sometimes vomiting [9,10]. Pelvic pain is often caused by the secretion of relaxin from the placenta, along with the enlargement of the lower abdomen, causing changes in the center of gravity of the body [11]. Pregnant women also experience distractedness, sudden drowsiness, and abdominal distension due to decreased intestinal peristalsis, in addition to sudden drowsiness caused by progesterone secretion [10]. Pregnancy-related anxiety is characterized by current and future concerns around pregnancy (e.g., childbirth, the baby’s health, and motherhood) [12]. Women often report problems with attention, concentration, and memory throughout pregnancy and the early puerperium. Poorer verbal recall, processing speed, and worse spatial recognition memory during pregnancy have also been reported [13]. Thus, health professionals must understand that physiological changes during pregnancy affect the mental and physical state of drivers.

The present study clarified that “tension and cramps in the lower abdomen”, “distractedness”, and “irritability” were independent, significant factors related to each event. To drive a vehicle safely, the appropriate cognitive, judgmental, and operational abilities are required. Specifically, the following functions are required: executive function for planning the entire driving action, attention function and visuospatial cognitive function for safe driving, information processing speed and emotional control, as well as operational knowledge and the ability to safely drive. The three factors that we identified could undermine the abilities required for safe driving. Therefore, pregnant women should avoid driving when they are experiencing common complaints of pregnancy, such as tension and cramps in the lower abdomen, distractedness, or irritability.

In general non-pregnant drivers, negative emotions such as “irritability”, “depressed mood”, and “distractedness” have been identified as significant factors involved in MVCs [14]. Our results indicated that distractedness and irritability were significant factors involved in MVCs and near-miss events involving pregnant women, in keeping with previous research findings.

Furthermore, the current study identified a novel finding that tension and cramps in the lower abdomen exerted the strongest influence on driving behavior in the event group of pregnant women. This complaint is due to acute changes in normal movement or contraction of the uterus, especially in mid- to late-term pregnancy. This result supports a previous study of pregnant women drivers that found that the risk of serious MCVs significantly increased during the early second trimester [3]. This common pregnancy complaint could also influence cognition and decision-making processes. Furthermore, lower abdomen tension and cramps also affect the movement of the lower limbs, which could delay pedal movements following cognition or decision-making processes. Although this symptom is reported by pregnant women themselves, midwives also notice it objectively by recognizing uterine contractions or abnormal bowel movements when taking a history and physical. Therefore, when tension and cramps in the lower abdomen are suspected in pregnant women, vehicle driving should be avoided. Additionally, informing pregnant women about the influence of common complaints on driving abilities is necessary, and self-care to stabilize these symptoms is required. According to a study of motor vehicle collision injuries between pregnant and non-pregnant women, interventions to prevent moderate and severe injuries in pregnant women were considered easier to develop than interventions for non-pregnant women because the collision severity was lower in pregnant women than in non-pregnant women [15]. As simple educational interventions produced by health professionals could help to reduce collisions in pregnant women, it is important to inform pregnant women that their first priority is to avoid driving when experiencing these symptoms to protect the life of both the mother and fetus.

This study has some limitations. First, because this study had a cross-sectional design, we did not compare the experience of events during pregnancy to those before pregnancy. Therefore, further study with a longitudinal design might be required. Second, this study was performed in Shiga Prefecture, with an approximate population of 1.4 million. Shiga Prefecture is a suburban area next to Kyoto, with different traffic patterns to other areas. In the future, similar research should be conducted throughout Japan and in multiple nations. Third, common complaints in pregnant women were assessed using 18 items that were selected because these symptoms are often observed in Japanese pregnant women. However, the items selected were based on previous studies that each focused on different complaints. Accordingly, in future research, we must ensure that items assessing common complaints in pregnancy are representative to improve the generalizability of the results. Fourth, our data included all pregnant women with short- and long-term driving histories across a broad gestational age range. Some of the complaints depended on gestational age, while driving ability also depended on driving history. As these values did not differ significantly between the event and non-event groups, these issues did not influence the conclusion. However, further analyses subcategorizing gestational age and driving history will be required in the future.

## 5. Conclusions

According to our results, health professionals need to discuss with their patients the physiological changes during pregnancy that can be related to driving ability. “Tension and cramps in the lower abdomen”, “distractedness”, and “irritability” were identified as independent factors influencing the occurrence of MVCs and near-miss incidents. Education about avoiding driving when experiencing these symptoms will improve both maternal and fetal safety.

## Figures and Tables

**Table 1:** Comparison of the basic characteristics between event and non-event groups.

		Total *n* = 626	Event Group *n* = 68	Non-Event Group *n* = 558	*p* Value
Age (years)	Median (IQR)	31.0 (28.0–35.0)	32.0 (29.0–35.0)	31.0 (28.0–35.0)	0.599
	Min–Max	18–44	22–41	18–44	
Height (cm)	Median (IQR)	159.0 (155.0–162.0)	158.0 (155.0–162.0)	159.0 (155.0–162.0)	0.628
	Min–Max	140.0–180.0	140.0–180.0	143.0–172.0	
Weight before pregnancy (kg)	Median (IQR)	51.0 (48.0–56.0)	51.5 (47.0–57.0)	51.0 (48.0–56.0)	0.977
	Min–Max	34.0–90.0	38.0–90.0	34.0–85.0	
Current weight (kg)	Median (IQR)	56.1 (51.7–62.0)	56.2 (51.0–63.0)	56.0 (52.0–62.0)	0.862
	Min–Max	34.0–88.0	44.5–82.0	34.0–88.0	
BMI before pregnancy	Median (IQR)	20.3 (19.0–22.2)	20.6 (19.4–22.4)	20.3 (18.9–22.1)	0.516
	Min–Max	14.0–37.0	16.4–37.0	14.0–33.2	
Gestational age (weeks)	Median (IQR)	27.0 (20.0–33.0)	29.0 (20.5–35.0)	27.0 (20.0–33.0)	0.111
	Min–Max	6–41	6–40	7–41	
Driving history (years)	Median (IQR)	11.0 (7.0–15.0)	12.0 (7.5–14.0)	11.0 (7.0–15.0)	0.740
	Min–Max	1–26	1–20	1–26	

IQR: interquartile range.

**Table 2:** Comparison of the frequency of each common complaint in pregnancy between event and non-event groups.

Complaint	Frequency	Event *n* = 68	%	Non-Event *n* = 558	%	*p* Value
Compression of the stomach or abdomen	Low	7	10.3	128	22.9	0.017
	High	61	89.7	430	77.1	
Nausea	Low	35	51.5	348	62.4	0.082
	High	33	48.5	210	37.6	
Frequent urination	Low	2	2.9	47	8.4	0.112
	High	66	97.1	511	91.6	
Tension and cramps in the lower abdomen	Low	10	14.7	173	31.0	0.005
	High	58	85.3	385	69.0	
Taut or tingling breasts	Low	35	51.5	317	56.8	0.402
	High	33	48.5	241	43.2	
Stiff shoulder	Low	23	33.8	230	41.2	0.241
	High	45	66.2	328	58.8	
Pelvic pain	Low	33	48.5	343	61.5	0.040
	High	35	51.5	215	38.5	
Back or lower back pain	Low	15	22.1	165	29.6	0.196
	High	53	77.9	393	70.4	
Fatigue and cramp in the lower limbs	Low	22	32.4	228	40.9	0.176
	High	46	67.6	330	59.1	
Whole-body malaise	Low	17	25.0	168	30.1	0.383
	High	51	75.0	390	69.9	
Strong drowsiness	Low	10	14.7	127	22.8	0.129
	High	58	85.3	431	77.2	
Irritability	Low	25	36.8	304	54.5	0.006
	High	43	63.2	254	45.5	
Depressed mood	Low	36	52.9	392	70.3	0.004
	High	32	47.1	166	29.7	
Distractedness	Low	24	35.3	309	55.4	0.002
	High	44	64.7	249	44.6	
Feeling of weakness	Low	39	57.4	352	63.1	0.357
	High	29	42.6	206	36.9	
Palpitations	Low	25	36.8	239	42.8	0.339
	High	43	63.2	319	57.2	
Hot flashes	Low	30	44.1	313	56.1	0.061
	High	38	55.9	245	43.9	
Itching	Low	38	55.9	347	62.2	0.313
	High	30	44.1	211	37.8	

**Table 3:** Relationship between event experiences and common complaints in pregnancy.

Complaints	Odds Ratio	95% Confidence Interval	*p* Value
Tension and cramps in the lower abdomen	2.414	1.198–4.868	0.014
Distractedness	1.849	1.072–3.189	0.027
Irritability	1.746	1.018–2.997	0.043

## Data Availability

The data presented in this study are available upon request from the corresponding author.
